# Supplementary material for: Common neural mechanisms supporting time judgements in humans and monkeys
Source: PeerJ. 2024 Nov 19;12:e18477. doi: 10.7717/peerj.18477 (PMC11583905; doi:10.7717/peerj.18477)
Supplement: Supplemental Information 1 [file peerj-12-18477-s001.pdf]

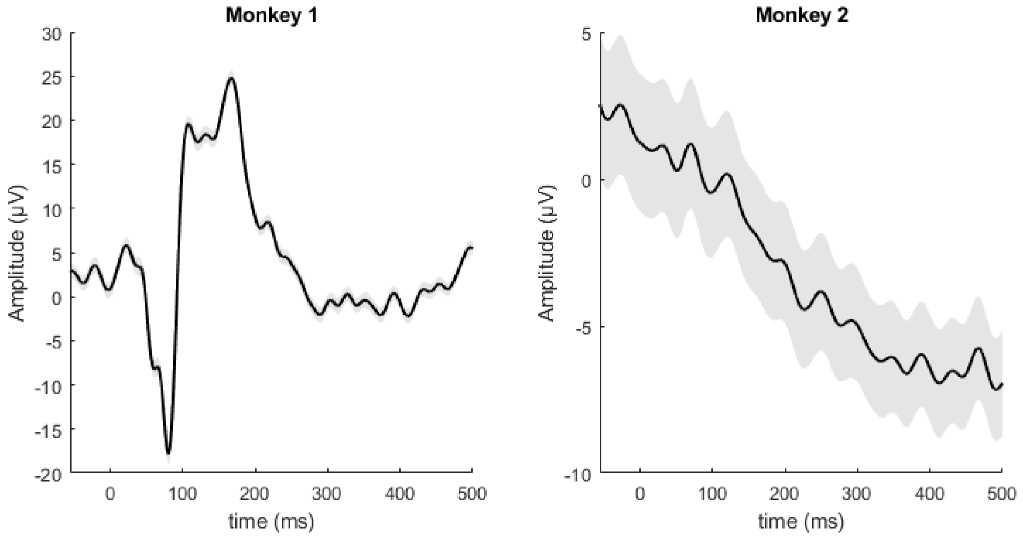

Supplementary Figure 1. Post-interval evoked potentials in monkeys. Event-related potentials in Monkey 1 (left panel) and Monkey 2 (right), shaded area showing SEM.

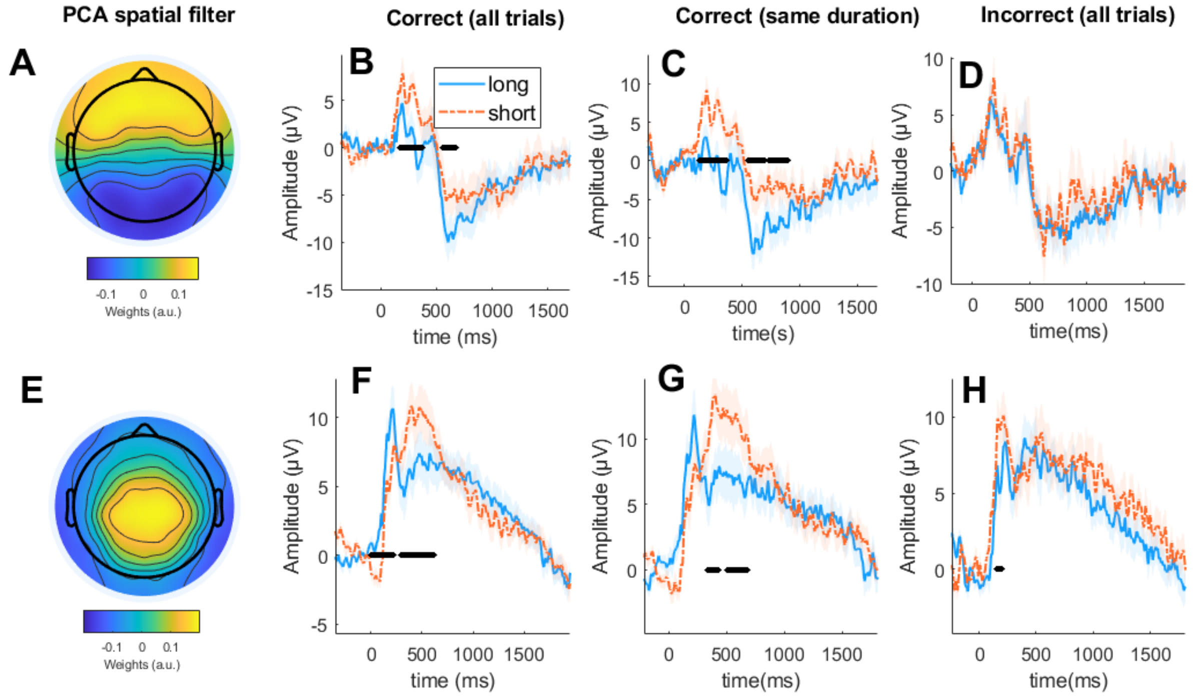

Supplementary Figure 2. Post-interval evoked potentials in humans (baseline corrected). Each row depicts the ERPs after a Group PCA-derived spatial filter is applied. (A) Topography of the spatial filters for the first principal component. (B) ERPs for “long” and “short” responses for correct trials. (C) ERPs for “long” and “short” responses for correct trials with the same objective (but differently perceived) duration. (D) ERPs for “long” and “short” responses for correct trials. (E-H) same as A-D for second component. Statistical significance at $p<0.025$ is marked with a black line, and 0 on the x-axis represents the offset of the stimulus whose duration was to be evaluated. Shaded area depicts SEM.

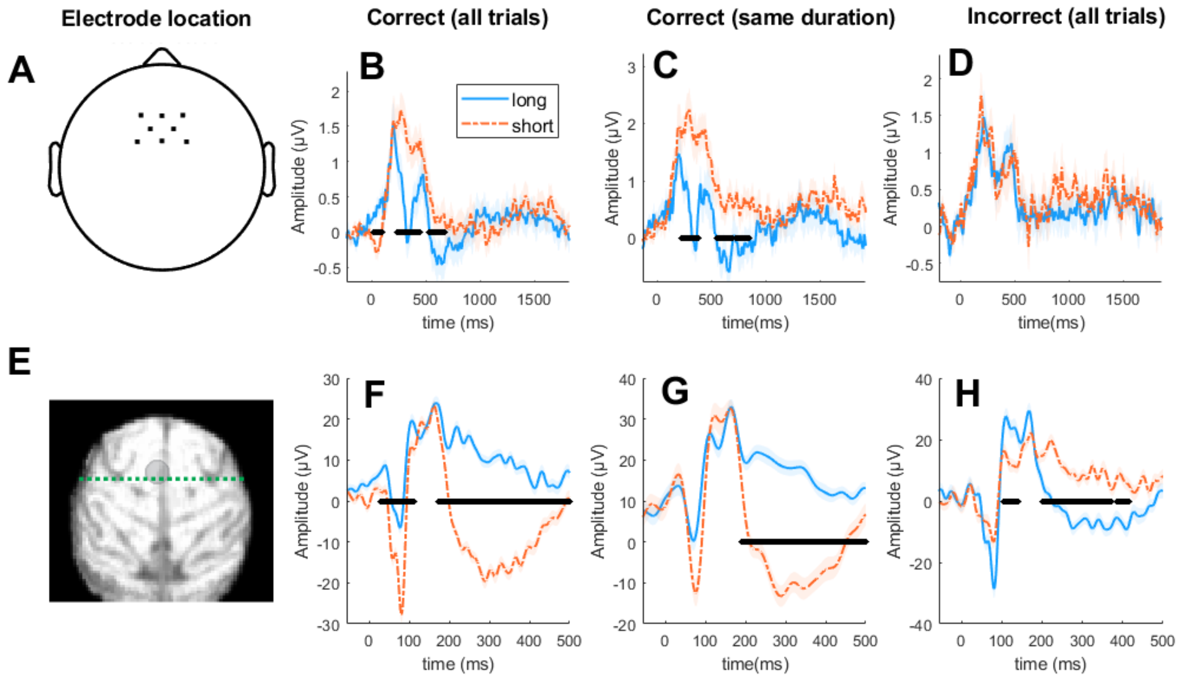

Supplementary Figure 3. Fronto-central post-interval evoked responses in human and non-human primates (baseline corrected). (A) Topography showing the location of electrode selection in humans. (B) ERPs for “short” (orange) and “long” responses (blue) in humans for correct trials. (C) ERPs for “short” (orange) and “long” responses (blue) in humans for correct trials with the same objective (but differently perceived) duration. D) ERPs for “short” (orange) and “long” responses (blue) in humans for incorrect trials. (E) Analysed recording sites in the monkey (pre-SMA). (F-H) Same as B-D but for the monkey. Statistical significance at $p<0.025$ is marked with a black line in each subplot, and 0 on the x-axis represents the offset of the stimulus whose duration was to be evaluated. Shaded area depicts SEM.
